# Essential amino acid-enriched whey enhances post-exercise whole-body protein balance during energy deficit more than iso-nitrogenous whey or a mixed-macronutrient meal: a randomized, crossover study

**DOI:** 10.1186/s12970-020-00401-5

**Published:** 2021-01-07

**Authors:** Jess A. Gwin, David D. Church, Adrienne Hatch-McChesney, Jillian T. Allen, Marques A. Wilson, Alyssa N. Varanoske, Christopher T. Carrigan, Nancy E. Murphy, Lee M. Margolis, John W. Carbone, Robert R. Wolfe, Arny A. Ferrando, Stefan M. Pasiakos

**Affiliations:** 1 Military Nutrition Division, U.S. Army Research Institute of Environmental Medicine, 10 General Greene Ave, Bldg. 42, Natick, MA 01760 USA; 2 Oak Ridge Institute for Science and Education, Belcamp, MD USA; 3 Department of Geriatrics, Donald W. Reynolds Institute on Aging, Center for Translational Research in Aging & Longevity, University of Arkansas for Medical Sciences, Little Rock, AR USA; 4 School of Health Sciences, Eastern Michigan University, Ypsilanti, MI USA

**Keywords:** Free-form amino acids, Muscle protein synthesis, Whole-body protein turnover, And energy restriction

## Abstract

**Background:**

The effects of ingesting varying essential amino acid (EAA)/protein-containing food formats on protein kinetics during energy deficit are undetermined. Therefore, recommendations for EAA/protein food formats necessary to optimize both whole-body protein balance and muscle protein synthesis (MPS) during energy deficit are unknown. We measured protein kinetics after consuming iso-nitrogenous amounts of free-form essential amino acid-enriched whey (EAA + W; 34.7 g protein, 24 g EAA sourced from whey and free-form EAA), whey (WHEY; 34.7 g protein, 18.7 g EAA), or a mixed-macronutrient meal (MEAL; 34.7 g protein, 11.4 g EAA) after exercise during short-term energy deficit.

**Methods:**

Ten adults (mean ± SD; 21 ± 4 y; 25.7 ± 1.7 kg/m^2^) completed a randomized, double-blind crossover study consisting of three, 5 d energy-deficit periods (− 30 ± 3% of total energy requirements), separated by 14 d. Whole-body protein synthesis (PS), breakdown (PB), and net balance (NET) were determined at rest and in response to combination exercise consisting of load carriage treadmill walking, deadlifts, and box step-ups at the end of each energy deficit using L-[^2^H_5_]-phenylalanine and L-[^2^H_2_]-tyrosine infusions. Treatments were ingested immediately post-exercise. Mixed-muscle protein synthesis (mixed-MPS) was measured during exercise through recovery.

**Results:**

Change (Δ postabsorptive + exercise to postprandial + recovery [mean treatment difference (95%CI)]) in whole-body (g/180 min) PS was 15.8 (9.8, 21.9; *P* = 0.001) and 19.4 (14.8, 24.0; *P* = 0.001) greater for EAA + W than WHEY and MEAL, respectively, with no difference between WHEY and MEAL. ΔPB was − 6.3 (− 11.5, − 1.18; *P* = 0.02) greater for EAA + W than WHEY and − 7.7 (− 11.9, − 3.6; *P* = 0.002) greater for MEAL than WHEY, with no difference between EAA + W and MEAL. ΔNET was 22.1 (20.5, 23.8; *P* = 0.001) and 18.0 (16.5, 19.5; *P* = 0.00) greater for EAA + W than WHEY and MEAL, respectively, while ΔNET was 4.2 (2.7, 5.6; *P* = 0.001) greater for MEAL than WHEY. Mixed-MPS did not differ between treatments.

**Conclusions:**

While mixed-MPS was similar across treatments, combining free-form EAA with whey promotes greater whole-body net protein balance during energy deficit compared to iso-nitrogenous amounts of whey or a mixed-macronutrient meal.

**Trial registration:**

ClinicalTrials.gov, Identifier no. NCT04004715. Retrospectively registered 28 June 2019, first enrollment 6 June 2019

## Introduction

Military personnel, weight-class athletes, and workers in arduous occupations, such as wildland firefighters, regularly experience periods of unavoidable energy deficit, which can degrade skeletal muscle and increase whole-body protein loss due, in part, to sustained negative whole-body protein balance and blunted muscle protein synthesis (MPS) [1–5]. Ingesting high-quality protein rich in essential amino acids (EAA) confers a potent anabolic stimulus during energy balance and, therefore, may offset muscle and whole-body protein loss if consumed during energy deficit [6–8]. However, recommendations for EAA/protein quantity and food format necessary to optimize both whole-body protein balance and MPS during energy deficit are not well described. Regarding EAA/protein quantity, we recently demonstrated that ingesting a high (0.30 g/kg) amount of free-form EAA during energy deficit enhances post-exercise whole-body protein balance to a greater extent than a standard (0.10 g/kg) amount consistent with current post-exercise protein ingestion recommendations [9].

The food format of the EAA/protein consumed dictates its quality and anabolic stimulus due to its constituent amino acid composition and its digestibility and absorption kinetics. These factors influence the amount and pattern of EAA entering peripheral circulation and therefore the substrate available to support whole-body protein turnover and MPS [10–13]. Mixed-macronutrient meals containing whole-food proteins are the predominant food format of EAA/protein consumed by the general population and by military personnel subsisting primarily on military rations during strenuous operations. However, to achieve an optimal amount of EAA/protein with mixed-macronutrient meals, individuals must consume more total food than if other more efficient EAA/protein formats were available. Consuming more food is often a logistical challenge during real world military training and operations [14, 15], ultra-endurance competitions, or wildfire suppression activities. Equally important, the anabolic stimulus provided by mixed-macronutrient meals may be suboptimal during these scenarios because the amino acid composition (EAA content), digestion and absorption rates, and subsequent increase in peripheral EAA concentrations are lower after consuming mixed-macronutrient meals than intact protein alone or free-form EAA. Whey protein is one alternative to mixed-macronutrient meals that is widely demonstrated to support protein anabolism [16]. Whey is comprised of approximately 40–50% EAA and is digested and absorbed quickly, resulting in a substantial increase in peripheral EAA concentrations. EAA ingested in free-form is another alternative to mixed-macronutrient meals, as it does not require digestion, is absorbed rapidly, and results in a rapid increase in peripheral EAA concentrations versus other EAA/protein food formats.

Combining intact protein with free-form EAA may be an advantageous anabolic formulation, in that it leverages the amino acid composition, digestion, and absorption kinetics of both EAA/protein formats to yield a robust and sustained increase in peripheral EAA concentrations. Churchward-Venne et al. [13] reported similar 3 h post-exercise myofibrillar MPS rates during energy balance after ingesting a low amount of whey enriched with EAA versus a standard amount of whey alone. To our knowledge, no studies have determined the effects of ingesting varying EAA/protein formats on protein kinetics during energy deficit. As such, we examined the effects of consuming three iso-nitrogenous EAA/protein delivery formats following exercise during energy deficit: a combined free-form EAA and whey protein mixture, whey protein alone, or a mixed-macronutrient meal. We hypothesized that a greater increase in peripheral EAA after ingesting EAA-enriched whey would enhance whole-body protein balance and MPS to a greater extent during energy deficit than whey or a mixed-macronutrient meal.

## Methods

### Participants

**Fig. 1:**
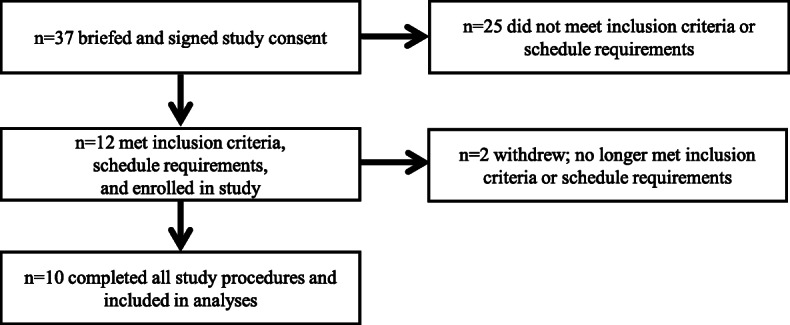
Volunteer enrollment and retention

Healthy (free of cardiovascular or metabolic disease as determined by a medical screening), young (18–25 y), non-obese (body mass index, < 30.0 kg/m^2^), resistance exercise-trained (≥ 2 sessions/week for previous 6 months) males and females were eligible to participate in this study. Volunteers were required to refrain from nonsteroidal anti-inflammatory medications, alcohol, nicotine products, caffeine, and dietary supplements throughout the study. Twelve male volunteers were enrolled in the study after providing informed, written consent (Fig. 1). One participant was withdrawn due to noncompliance before data collection and one participant was withdrawn due to personal reasons after completing one energy deficit testing period. Therefore, 10 volunteers completed all study procedures and were included in the final analyses (Table 1). This study was approved by the U.S. Army Medical Research and Development Command Institutional Review Board and registered at www.clinicaltrials.gov (NCT04004715). Investigators adhered to the policies for protection of human subjects as prescribed in the U.S. Department of Defense Instruction 3216.02, and the research was conducted in adherence with the provisions of 32 Code of Federal Regulations Part 219.

**Table 1:** Baseline participant characteristics^1^

	*n* = 10
Age (y)	21 ± 4
Sex (m/f)	8/2
Body Mass (kg)	77.6 ± 9.1
Height (cm)	173.5 ± 8.9
Body Mass Index (kg/m^2^)	25.7 ± 1.7
VO_2peak_ (mL/kg/min)	46.4 ± 6.4
3RM Deadlift (kg)	127.7 ± 25.4
Estimated 1RM Deadlift (kg)	137.4 ± 27.4

^1^Values are means ± SD

### Experimental design

Volunteers underwent a randomized, crossover study consisting of three, 5 d controlled, diet-induced energy deficits (− 30% of total energy requirements), each separated by a 14 d washout. Immediately following each energy deficit, stable isotope infusion studies were used to determine whole-body protein synthesis (PS), protein breakdown (PB), and net balance (NET) in response to post-exercise ingestion of an EAA-enriched, low dose of whey protein isolate (EAA + W; 35 g protein) or iso-nitrogenous amounts of whey protein isolate (WHEY) or protein in a mixed-macronutrient meal (MEAL). Mixed-MPS was also assessed for the entire exercise plus postprandial recovery period. Volunteers were provided an individualized 3 d run-in, weight-maintaining diet immediately preceding each energy deficit diet to limit any potential confounding effects of pre-study diet and to maintain consistency with our prior research [9, 17–19]. To limit the effects of previous exercise on protein turnover [20], routine exercise was prohibited throughout the diet interventions. Treatment order was randomized to avoid bias using a random numbers generator (https://www.randomizer.org). Treatments were semi-blinded such that all volunteers and study staff were blinded to the protein beverages (EAA + W and WHEY), excluding a designated staff member who developed the treatment code and prepared the treatments, but was not involved in primary outcome data analysis or interpretation.

### Anthropometrics

Height was measured in duplicate to the nearest 0.1 cm using a stadiometer (Seritex, Inc., Carlstadt, NJ, USA) at baseline. Fasted (overnight, ≥ 8 h), nude body weights were measured to the nearest 0.1 kg after a void using a digital scale (Taylor Precision Products, Oak Brook, IL, USA) at baseline, daily throughout each intervention, and every third day during the 14 d washouts. Fat mass and fat-free mass [total mass – (fat mass + bone mass)] were determined using dual energy X-ray absorptiometry (DXA; Lunar iDXA, Ge Healthcare, Madison, WI, USA) at baseline and on the fifth day of each energy deficit, after an ≥8 h overnight fast and void, to characterize changes in body composition.

### Diet intervention

Pre-study dietary intake and physical activity levels were assessed using 3 d diet and activity records (2 weekdays, 1 weekend day). Dietary records were analyzed (Food Processor SQL, v.11.3.2) and total daily energy requirements for the 3 d run-in diets were determined using the average of the Harris-Benedict [21] and Mifflin-St Jeor [22] equations, multiplied by 1.3 to account for activities of daily living and diet-induced thermogenesis. Registered Dietitians developed individualized menus (Food Processor SQL, v.11.3.2; ESHA Research, Salem, OR, USA) consisting primarily of military combat rations (Meal, Ready-to-Eat; menu 37; Ameriqual, Evansville, IN, USA), supplemented with commercial products (e.g., frozen sandwiches, yogurt, snack foods). To be consistent with our previous work [9], dietary protein was provided at 1.6 g/kg/d, carbohydrate comprised 50–55% of total energy, and fat provided the remaining energy. The 30% energy deficit was achieved by reducing carbohydrate and fat intakes while maintaining protein intake at 1.6 g/kg/d. All foods and beverages were weighed to the nearest 0.1 g and distributed to the volunteers at the start of each run-in and energy deficit diet. Volunteers were instructed to consume all of the provided foods and beverages and return the empty packaging. Any uneaten foods or beverages were weighed and accounted for in reported intakes. Water was allowed ad libitum. Volunteers were instructed to return to their pre-study dietary habits and physical activity patterns during the 14 d washouts. Dietary habits and physical activity were recorded every third day during the washouts using 24 h diet and activity records.

### Stable isotope infusion studies

**Fig. 2:**
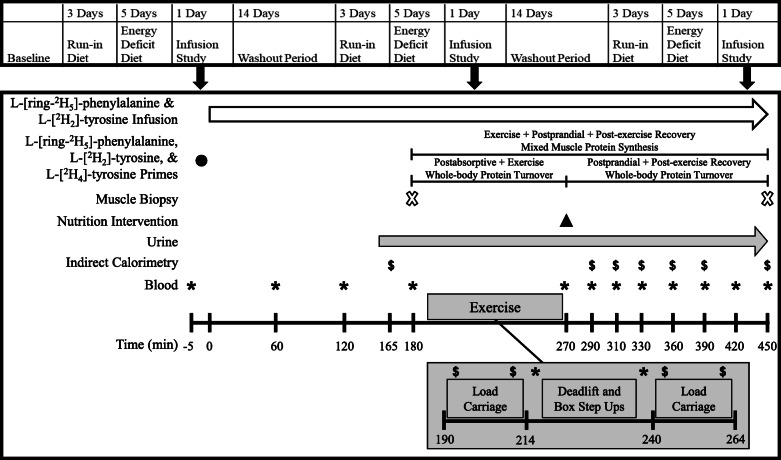
Schematic of the infusion studies. Muscle biopsy and blood samples were used in combination with primed, constant infusions of L-[^2^H_5_]-phenylalanine and L-[^2^H_2_]-tyrosine to determine the effects of EAA + W, WHEY, or MEAL ingestion on whole-body protein turnover following whole-body exercise as well as mixed muscle protein synthesis throughout an exercise and recovery period during energy deficit

Stable isotope infusion studies were conducted the morning (after ≥8 h overnight fast) following each 5 d energy deficit to determine whole-body protein turnover and mixed-MPS (Fig. 2). Intravenous catheters were placed in the antecubital space or forearm of each arm for the continuous isotope infusions and serial blood draws. The arm used for serial blood draws was warmed using heating pads so that the sampled blood reflected arterialized blood [23]. Following the baseline blood sample, primed, constant infusions of L-[ring-^2^H_5_]-phenylalanine and L-[3,3-^2^H_2_]-tyrosine were started and maintained for the next 450 min. A priming dose of L-[ring-^2^H_4_]-tyrosine was administered at the start of the infusion to achieve isotopic equilibrium of L-[ring-^2^H_4_] tyrosine enrichment derived from L-[ring-^2^H_5_]-phenylalanine. All isotopes were purchased from Cambridge Isotope Laboratories (Andover, MA, USA) and the preparations were constituted by a licensed pharmacist and certified sterile and pyrogen-free (Johnson Compounding and Wellness, Waltham, MA, USA).

Two muscle biopsies were collected from the vastus lateralis using a single incision on one leg during each infusion study to assess mixed-MPS. All muscle biopsies were performed under sterile conditions using a 5 mm Bergstrom biopsy needle. All tissue was blotted dry of blood and all visible fat and connective tissue was removed before the tissue was frozen in liquid nitrogen and stored at -80 °C until analysis. The first muscle biopsy was performed 10 min prior (180 min after infusion initiation) to a bout of whole-body exercise. The exercise bout consisted of 24 min of load carriage (LC) followed by 18 min of alternating trap bar deadlifts and box step-ups followed by another 24 min of LC. Volunteers were given 4 min of rest before and after the bout of deadlifts and step-ups. All LC was performed by walking on a treadmill while wearing a weighted pack equivalent to 30% of each individual’s baseline body mass. Speed and grade were adjusted throughout the LC to achieve 1 min intervals of low to moderate intensity (55 ± 5%) and moderate to vigorous intensity (70 ± 5%) work based on V̇O_2peak_ determined at baseline and confirmed during each washout period. If the volunteer was unable to complete the prescribed workload, the speed of the treadmill was reduced until the participant could complete the work. Every effort was made to match LC bouts between infusion studies and the bouts were nearly identical between all trials for all volunteers. For each set of trap bar deadlifts and box step-ups, volunteers completed 5 repetitions of deadlift immediately followed by 16 step-ups (8 per leg) totaling to ~ 1 min of work. The volunteers then rested for 1 min before completing the next set. In total, 9 sets were performed. Volunteers were supervised to ensure accurate repetition counts and safe lifting form. The weight on the trap bar was prescribed at 70% of the individuals’ estimated 1 repetition maximum (RM) derived from a predetermined 3RM [24, 25] and matched between all trials. All volunteers completed a familiarization session during baseline to confirm the intensities of the LC exercise prescription and the ability of each individual to complete the entire exercise bout. Within ~ 5 min of completing the exercise bout (270 min), volunteers consumed either EAA + W dissolved in 200 mL of water as a bolus (proprietary free-form EAA and whey protein blend; The Amino Company LLC, Lewes, DE, USA),bolus (proprietary free-form EAA and whey protein blend; The Amino Company LLC, Lewes, DE, USA), WHEY dissolved in 200 mL of water as a bolus (BiPro Elite Whey Protein Isolate; BiPro USA, Eden Prairie, MN, USA) or MEAL (Chili and Beans Entrée, Meal, Ready-to-Eat; menu 37; Ameriqual, Evansville, IN, USA; Table 2). Volunteers were given an additional 300 mL and 500 mL of water to consume with the beverages and MEAL, respectively. The study treatments and additional water were consumed within 5 min. Thereafter, volunteers rested for the remaining 180 min recovery period while blood samples were continually collected until a final biopsy was performed at 450 min (Fig. 2). For the second biopsy within a given day, the biopsy needle was angled away from the previous sampling location by ~ 5 cm to reduce the chance of sampling from a pre-biopsied area and to avoid local inflammation [26–28]. The biopsied leg alternated between infusion studies and a new incision was made for the third infusion study ~ 3 to 5 cm from the first infusion study incision [27].

**Table 2:** Nutrient profiles of the study treatments^1^

	EAA + W	WHEY	MEAL
Protein (g)	34.7	34.7	34.7
Total EAA (g)	24.0	18.7	11.4
Carbohydrate (g)	5.2	1.9	60.1
Fat (g)	1.4	0.4	20.8
Total Energy (kcal)	172.6	150.3	566.9
Serving Size of Product (g)	46.3	39.8	561.3
*EAA Composition (% of EAA)*			
*histidine*	*7.7*	*3.9*	*6.7*
*isoleucine*	*11.6*	*11.6*	*11.5*
*leucine*	*20.2*	*25.7*	*20.6*
*lysine*	*18.2*	*22.1*	*16.5*
*methionine*	*3.7*	*4.7*	*4.7*
*phenylalanine*	*10.7*	*7.2*	*12.7*
*threonine*	*14.8*	*9.9*	*10.8*
*tryptophan*	*1.2*	*5.0*	*2.9*
*valine*	*11.8*	*9.9*	*13.6*

^1^The macronutrient and amino acid profile of EAA + W, WHEY, and MEAL were confirmed by chemical analysis (Eurofins Food Integrity and Innovation, Madison, WI)

### Analytical procedures

Plasma and muscle processing were consistent with our previous work [9]. Plasma was precipitated with 125 μL of 10% sulfosalicylic acid (SSA), centrifuged, and the supernatant was used to determine EAA concentrations using the internal standard technique and liquid chromatography with tandem mass spectrometry (LCMS: QTrap 5500 MS;AB Sciex, Foster City, CA) [29]. The intra-assay coefficient of variation was 1.01, 1.36, 1.26, 2.80, 1.79, 0.99, 1.09, 1.13, 1.84, and 0.88% for threonine, valine, methionine, isoleucine, leucine, tryptophan, phenylalanine, histidine, lysine, and tyrosine, respectively. Phenylalanine and tyrosine enrichments were measured using the tert-butyldimethylsilyl derivative and gas chromatography-mass spectrometry (models 7890A/5975; Agilent Technologies, Santa Clara, CA) [30, 31]. Ions of mass-to-charge ratio of 234, 235, and 239 for phenylalanine and of 466, 467, 468, and 470 for tyrosine were monitored with electron impact ionization and selective ion monitoring. Serum insulin concentrations were measured using a Siemens Immulite 2000XPI (Siemens Medical Solutions USA, Inc., Malvern, PA). The intra-assay coefficient of variation was 3.84% for insulin. Muscle samples were weighed and tissue proteins were precipitated with 0.5 mL of 4% SSA. Next the samples were homogenized, centrifuged, and the muscle pellet (bound protein) was washed, dried, and hydrolyzed in 0.5 mL of 6 N HCl at 105 °C for 24 h. Mixed-muscle-bound protein enrichments were determined as described above for plasma enrichments.

### Whole-body PS, PB, and NET and mixed-MPS calculations

**Fig. 3:**
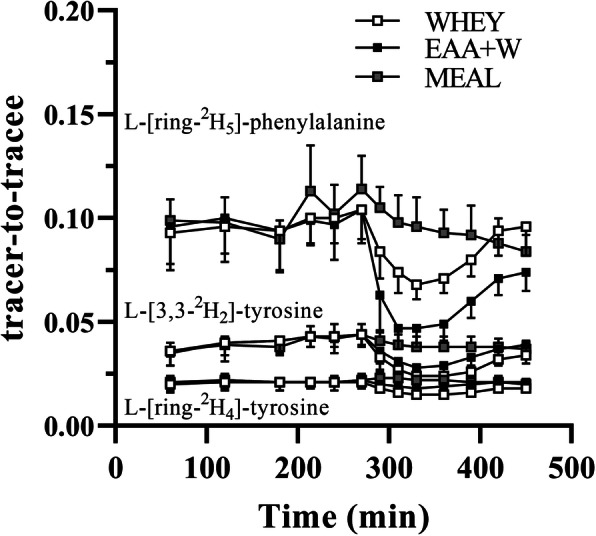
Mean ± SD (*n* = 10). Stable-isotope enrichments during the infusion studies

Whole-body PS and PB rates were calculated based on the determinations of the rate of appearance (Ra) into the plasma of phenylalanine and tyrosine and the fractional Ra of endogenous tyrosine derived from phenylalanine [19, 32]. Total Ra over time after intervention were calculated to avoid the complication of calculating non-steady state kinetics. The phenylalanine (Phe) and tyrosine (Tyr) plasma enrichment areas under the curve (AUC) were calculated from start to 450 min (Fig. 3). Whole-body protein turnover was calculated by dividing kinetic values of phenylalanine by its fractional contribution to protein. For calculations of whole-body PB rate, contribution from exogenous Phe and Tyr were subtracted from total Ra. The following equations were used to calculate whole-body PS, PB, and NET [9]:

$$ \mathsf{Total}\ \mathsf{plasma}\ {\mathsf{R}}_{\mathsf{a}}=\mathsf{F}/\mathsf{E} $$

$$ \mathsf{Fractional}\ {\mathsf{R}}_{\mathsf{a}}\ \mathsf{of}\ \mathsf{Tyr}\ \mathsf{from}\ \mathsf{Phe}={\mathsf{E}}_{\mathsf{Tyr}\ \mathsf{M}+\mathsf{4}}/{\mathsf{E}}_{\mathsf{Phe}\ \mathsf{M}+\mathsf{5}} $$

$$ \mathsf{Phe}\ \mathsf{hydroxylation}=\mathsf{fractional}\ {\mathsf{R}}_{\mathsf{a}}\ \mathsf{of}\ \mathsf{Tyr}\ \mathsf{from}\ \mathsf{Phe}\ \mathsf{x}\ {\mathsf{R}}_{\mathsf{a}}\ \mathsf{Tyr} $$

$$ \mathsf{PS}=\left[\left({\mathsf{R}}_{\mathsf{a}}\ \mathsf{Phe}-\mathsf{Phe}\ \mathsf{hydroxylation}\right)\ \mathsf{x}\ \mathsf{25}\right] $$

$$ \mathsf{Exogenous}\ {\mathsf{R}}_{\mathsf{a}}\ \mathsf{Phe}=\left(\mathsf{Ingested}\ \mathsf{Phe}\ \mathsf{x}\ \mathsf{digestibility}\right)-\mathsf{Phe}\ \mathsf{hydroxylation}\ \mathsf{above}\ \mathsf{basal} $$

$$ \mathsf{PB}=\left[\left(\mathsf{Total}\ {\mathsf{R}}_{\mathsf{a}}\ \mathsf{Phe}-\mathsf{Exogenous}\ {\mathsf{R}}_{\mathsf{a}}\ \mathsf{Phe}\right)\ \mathsf{x}\ \mathsf{25}\right] $$

$$ \mathsf{NET}=\mathsf{PS}-\mathsf{PB} $$

where E is enrichment of respective tracers at plateau and expressed as tracer-to-tracee ratio (TTR) or mole percent excess (MPE), calculated as TTR/(TTR + 1). TTR was used for calculations of PB, whereas MPE was used for calculations of PS. F is respective tracer infusion rate into a venous side: F_Phe_ for phenylalanine tracer. E_Tyr M + 4_ and E_Phe M + 5_ are plasma enrichments of tyrosine and phenylalanine tracers at M + 4 and M + 5 relative to M + 0, respectively. In the fed state, fractional R_a_ of Tyr from Phe was divided by 0.8 to account for hepatic dilution [33]. The correction factor of 25 is for conversion of phenylalanine values to total protein based on the assumption that the contribution of phenylalanine to skeletal muscle protein is 4% (100/4 = 25) [34]. Phe is the amount of exogenous phenylalanine (g) that appeared in circulation, which was calculated as total amount of Phe provided (in the postprandial period only), based on the assumption that 99.5, 99, and 94% of the ingested Phe was absorbed for the EAA + W, WHEY, and MEAL, respectively [35, 36]. Phe hydroxylation is the R_a_ of tyrosine derived via hydroxylation of phenylalanine. Change in whole-body PS, PB, and NET were also examined normalized to EAA intake by dividing PS, PB, and NET values by the g of EAA provided in the EAA + W (24 g), WHEY (18.7 g), and MEAL (11.4 g) treatments to determine the synthetic stimulus per g of EAA consumed.

The plasma Phe and Tyr enrichment curves (Fig. 3) describe the physiological perturbations resulting from the digested amino acids in each treatment. Although the enrichment perturbations nearly returned to plateau values for both beverages, the infusion was too short for plasma enrichments to completely return to pre-meal values. We considered these perturbations when calculating the Phe enrichment AUCs, and in turn, PS, PB, and NET. Calculations were conducted using two approaches: first by using the measured values at 450 min, and second by imputing values for 450 min representative of a return to plateau (i.e., time point 270 min). A comparison of the results revealed nearly identical NET, PS, and PB values, most likely because by 450 min the majority of the postprandial physiological response had already been characterized. Therefore, the qualitative findings are consistent with using the measured/physiological values. We acknowledge that the postprandial infusion was also too short to characterize full digestion and absorption of MEAL. Since the whole-body protein turnover calculations were executed under the assumption that all exogenous Phe is digested and absorbed during the measurement period, the total R_A_ is artificially increased resulting in an underestimation of PB for MEAL. However, the intent of the current study was to evaluate early, acute postprandial responses and the data we present reflect the whole-body protein turnover that occurs within this period. All findings should be interpreted within this context.

The precursor-product model was used to determine mixed-MPS (i.e., fractional synthetic rate) [37]:

$$ \mathsf{Mixed}-\mathsf{MPS}\ \left(\%/\mathsf{h}\right)=\left[\left({\mathsf{E}}_{\mathsf{BP2}}-{\mathsf{E}}_{\mathsf{BP1}}\right)/\left({\mathsf{E}}_{\mathsf{p}}\right)\right]\times \mathsf{60}\times \mathsf{100} $$

where E_BP1_ and E_BP2_ are the enrichments of bound L-[ring-^2^H_5_]-phenylalanine in muscle collected pre and post-exercise (450 min – 180 min). The precursor enrichment (E_p_) is the calculated AUC for L-[ring-^2^H_5_]-phenylalanine enrichment in the plasma extracellular pool from 180 min to 450 min to accurately reflect blood perturbations, which is consistent with our previous work [9] and others [38]. Factors 60 and 100 were used to express mixed-MPS as percent per hour. Mixed-MPS was also normalized to energy intake by dividing mixed-MPS by the energy provided in the EAA + W (150.3 kcal), WHEY (172.6 kcal), and MEAL (566.9 kcal) treatments to determine the synthetic stimulus per kcal consumed.

### Statistical analysis

Previous research examining NET [19] was used to determine statistical power and sample size. An expected mean difference of 18.9 g/180 min in NET between the EAA + W, WHEY and MEAL treatments, a SD of 2.0 g/180 min, and an α of 0.05, were used to detect differences with a minimum of 10 volunteers. This sample size also provided ≥85% power to detect differences in mixed-MPS between study treatments based on an expected mean difference of 0.01%/h between the EAA + W, WHEY, and MEAL treatments, a SD of 0.01%/h, and an α of 0.05 [38–40].

The primary outcomes for this study were whole-body protein turnover responses to ingesting EAA + W, WHEY, and MEAL and mixed-MPS responses across the exercise plus postprandial recovery period. Secondary outcomes included EAA, leucine, phenylalanine, tyrosine, and insulin concentrations over time and incremental area under the curve (iAUC) following EAA + W, WHEY, and MEAL ingestion.

Linear mixed models, with participant treated as a random effect, were used to determine the effects of treatment (EAA + W, WHEY, and MEAL), condition (postabsorptive and postprandial), and their interaction (treatment-by-condition) on whole-body protein kinetics and phenylalanine hydroxylation. A one-way repeated measures analysis of variance (ANOVA) was used to determine the effects of treatment (EAA + W, WHEY, and MEAL) on change in (Δ postabsorptive + exercise/postprandial + recovery) whole-body protein kinetics and mixed-MPS. Where the magnitude of difference between treatments or conditions is presented, the value of the mean difference (95% CI) is reported. Two-way repeated measures ANOVA were used to determine the effects of treatment (EAA + W, WHEY, and MEAL), time (min), and their interaction (treatment-by-time) on plasma EAA, leucine, phenylalanine, tyrosine, and insulin concentrations. EAA, leucine, phenylalanine, tyrosine, and insulin were also calculated using iAUC [41] and one-way repeated measures ANOVA were used to evaluate whether iAUC differed between EAA + W, WHEY, and MEAL. One-way repeated measures ANOVA were used to assess potential changes in body composition (i.e., fat-free mass and fat mass at baseline and the end of each energy deficit period), body mass (i.e., day 1 of each run-in and day of each infusion study), change in body mass during each deficit (i.e., day 3 of each run-in minus day of each infusion study), and exercise intensity of LC during each infusion study. Paired samples t-test were used to determine decreases in body mass during each energy deficit (i.e., day 3 of each run-in and day of each infusion study). Bonferroni post hoc comparisons were used if main or interaction effects were significant. Data for all primary outcomes exhibited normality as assessed by Shapiro-Wilk, therefore parametric statistics were used. Sphericity was assessed for all data using Mauchly’s Test of Sphericity and when appropriate, the Huynh-Feldt correction was applied. Trial order effects were examined using a linear mixed model for whole-body PS, PB, and NET and a one-way repeated measures ANOVA for MPS and confirmed no order effects. All statistical analyses were performed with IBM SPSS software (version 26; IBM Corp. Armonk, NY, USA). Significance was set at *P* < 0.05 and data are presented as means ± SD.

## Results

Body mass on day 1 of each run-in diet was the same (*P* = 0.76; Table 3). Body mass was reduced during the energy deficit in all treatments (all, *P* = 0.01) and the magnitude of reduction was the same between treatments (*P* = 0.81). Therefore, body mass on the day of the infusion studies was the same between treatments (*P* = 0.92). There was a main effect of time point for fat-free mass (*P =* 0.044), however post-hoc comparisons indicated no differences in fat-free mass measured at baseline and at the end of each energy deficit or between each energy deficit (all, *P >* 0.1). Fat mass was the same at baseline and at the end each energy deficit and between each energy deficit (*P* = 0.73). Run-in and energy deficit diet characteristics as well as the magnitude of energy deficit incurred did not differ between treatments (each variable, *P* > 0.1; Table 4).

**Table 3:** Body composition^1^

	Baseline	EAA + W Run-in Day 1	WHEY Run-in Day 1	MEAL Run-in Day 1	EAA + W Infusion Study	WHEY Infusion Study	MEAL Infusion Study
Body mass (kg)	77.6 ± 9.1	78.5 ± 9.0^a^	78.7 ± 8.8^a^	78.3 ± 8.4^a^	76.8 ± 9.4^b^	76.9 ± 8.4^b^	76.8 ± 8.5^b^
∆ Body mass (kg)	---	---	---	---	-1.1 ± 0.5	-1.2 ± 0.7	-1.3 ± 1.2
Fat-free mass (kg)	55.6 ± 8.9	---	---	---	54.6 ± 9.1	54.5 ± 8.3	54.9 ± 8.6
Fat mass (kg)	19.2 ± 5.6	---	---	---	19.3 ± 5.6	19.4 ± 6.2	19.0 ± 5.5

^1^Values are means ± SD. ∆ defined as change from run-in day 3 and the infusion study day. Different lowercase letter superscripts indicate difference between body mass on run-in day 1 and on the infusion study day within the same treatment (all, *P* = 0.01)

**Table 4:** Dietary intake during each intervention period ^1^

	Baseline^2^	EAA + W Run-in	WHEY Run-in	MEAL Run-in	EAA + W Energy deficit	WHEY Energy deficit	MEAL Energy deficit
*Absolute Intake (kcal/d or g/d)*							
Energy	2875.5 ± 801.8	2325.6 ± 247.9	2351.2 ± 248.0	2316.2 ± 254.4	1624.4 ± 211.2	1623.7 ± 202.6	1623.1 ± 208.9
Protein	118.1 ± 31.4	126.4 ± 16.5	128.1 ± 15.8	128.1 ± 15.8	125.7 ± 16.3	125.3 ± 15.1	126.2 ± 15.7
Carbohydrate	319.4 ± 86.8	312.3 ± 31.3	313 ± 31.3	309.3 ± 31.7	190.5 ± 28.5	190.8 ± 27.1	190.4 ± 27.4
Fat	104.0 ± 35.5	67.5 ± 8.3	69.5 ± 8.3	67.2 ± 8.8	41.8 ± 4.2	42.0 ± 4.7	41.6 ± 4.6
*Relative Intake* *(kcal* ^*3*^*/kg/d or g/d)*							
Energy	36.9 ± 8.1	29.7 ± 1.8	30.1 ± 1.4	29.6 ± 1.6	21.0 ± 1.5	21.0 ± 1.3	21.0 ± 1.3
Protein	1.6 ± 0.6	1.6 ± 0.1	1.6 ± 0.0	1.6 ± 0.1	1.6 ± 0.1	1.6 ± 0.1	1.6 ± 0.1
Carbohydrate	4.1 ± 1.0	4.0 ± 0.2	4.0 ± 0.2	4.0 ± 0.2	2.5 ± 0.2	2.5 ± 0.2	2.5 ± 0.2
Fat	1.3 ± 0.4	0.9 ± 0.1	0.9 ± 0.1	0.9 ± 0.1	0.5 ± 0.0	0.5 ± 0.0	0.5 ± 0.0
*Energy Intake (%)*							
Protein	17 ± 4	22 ± 1	22 ± 1	22 ± 1	31 ± 1	31 ± 1	31 ± 2
Carbohydrate	46 ± 7	54 ± 1	53 ± 1	53 ± 1	47 ± 1	47 ± 1	47 ± 1
Fat	33 ± 3	27 ± 1	26 ± 1	26 ± 1	23 ± 1	23 ± 2	23 ± 1
*Energy Deficit (%)*							
Energy	–	–	–	–	30.3 ± 3.3	31.0 ± 3.6	30.0 ± 3.4

^1^ Values are means ± SD (*n* = 10). Dietary intake was directly measured during each 3 d run-in and 5 d energy deficit diet

^2^ Estimated intakes derived from 3 d dietary recalls

^3^ 1 kcal = 4184 joules

### Exercise intensity

Exercise characteristics are listed in Table 5. VO_2peak_ did not differ between baseline, washout one, and washout two (*P* = 0.11; Table 5). Exercise intensity, as measured by VO_2_, during the LC bouts did not differ between treatments (*P* = 0.82).

**Table 5:** VO_2peak_ and exercise characteristics^1^

	Baseline	Washout 1	Washout 2	EAA + W Infusion day	WHEY Infusion day	MEAL Infusion day
VO_2peak_ (mL/kg/min)	46.4 ± 6.4	46.7 ± 5.9	45.3 ± 6.3	---	---	---
Load Carriage VO_2_ (mL/kg/min)	---	---	---	22.0 ± 2.8	22.1 ± 2.5	22.1 ± 2.8
70% of Estimated 1RM	212.0 ± 41.5	---	---	212.0 ± 41.5	212.0 ± 41.5	212.0 ± 41.5

^1^Values are means ± SD

### Whole-body protein turnover and mixed-MPS

**Fig. 4:**
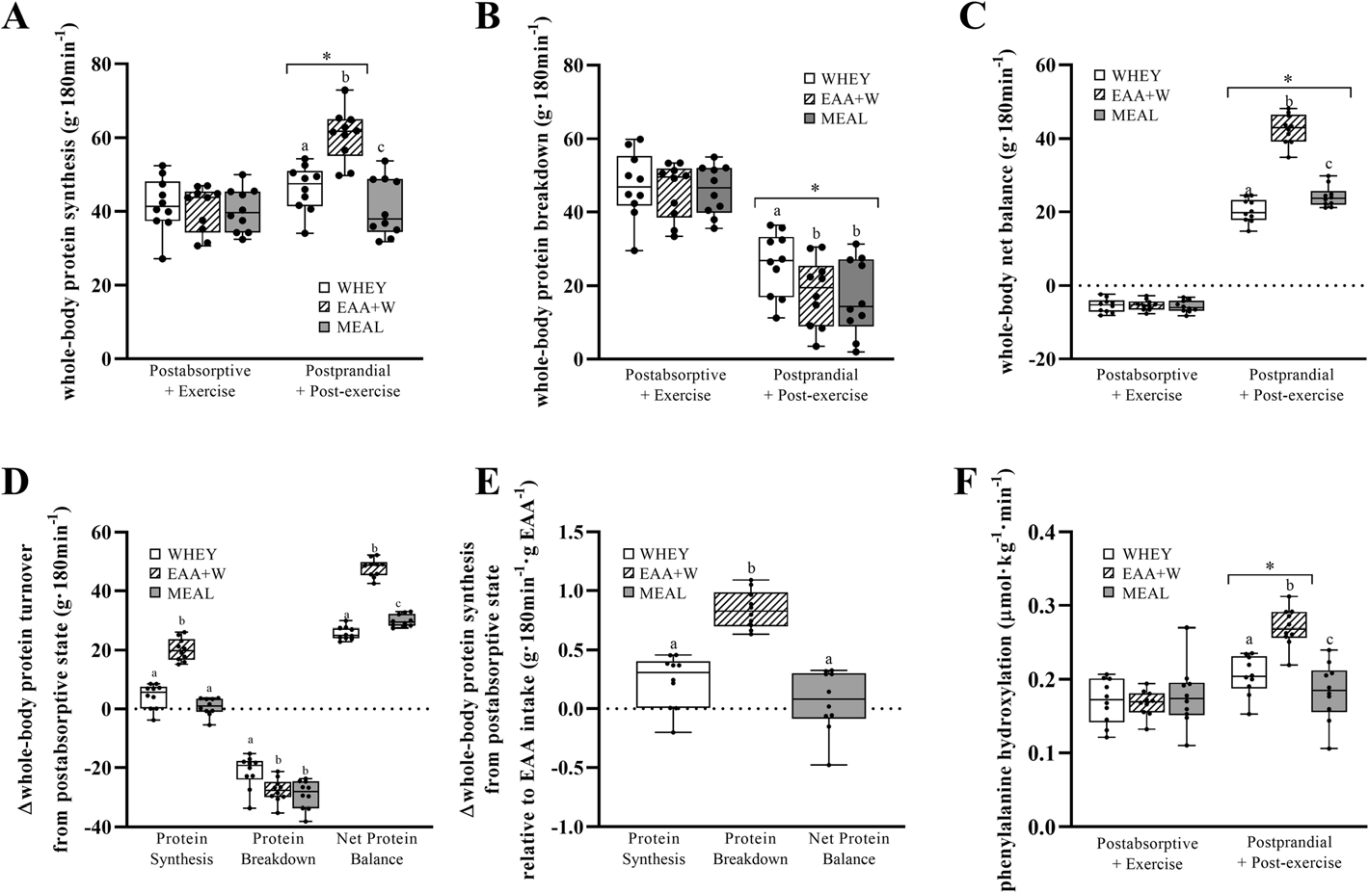
**a**: Mean ± SD (*n* = 10). Postabsorptive plus exercise and postprandial post-exercise recovery whole-body protein synthesis after WHEY, EAA + W, and MEAL intake during energy deficit. *indicates post hoc difference between postabsorptive and postprandial within the same treatment (*P* = 0.001) and different lowercase letters indicate post hoc difference between treatments within the same condition (both, *P* < 0.01). **b**: Mean ± SD (*n* = 10). Postabsorptive plus exercise and postprandial post-exercise recovery whole-body protein breakdown after WHEY, EAA + W, and MEAL intake during energy deficit. *indicates post hoc difference between postabsorptive and postprandial within the same treatment (*P* = 0.011) and different lowercase letters indicate post hoc difference between treatments within the same condition (both, *P* = 0.001). **c**: Mean ± SD (*n* = 10). Postabsorptive plus exercise and postprandial post-exercise recovery whole-body net balance after WHEY, EAA + W, and MEAL intake during energy deficit. *indicates post hoc difference between postabsorptive and postprandial within the same treatment (*P* = 0.001) and different lowercase letters indicate post hoc difference between treatments within the same condition (both, *P* = 0.001). **d**: Mean ± SD (*n* = 10). Change in postabsorptive plus exercise and postprandial post-exercise recovery whole-body protein turnover after WHEY, EAA + W, and MEAL intake during energy deficit. Different lowercase letters indicate difference between treatments within the protein synthesis, protein breakdown, and net balance measures (all, *P* < 0.05). **e**: Change in postabsorptive plus exercise and postprandial post-exercise recovery whole-body protein synthesis relative to EAA intake after WHEY, EAA + W, and MEAL intake during energy deficit. Different lowercase letters indicate difference between treatments (all, *P* < 0.05). **f**: Mean ± SD (*n* = 10). Postabsorptive plus exercise and postprandial post-exercise recovery phenylalanine hydroxylation after WHEY, EAA + W, and MEAL intake during energy deficit. *indicates post hoc difference between postabsorptive and postprandial within the same treatment (*P* = 0.001) and different lowercase letters indicate post hoc difference between treatments within the same condition (both, *P* = 0.001)

Postabsorptive whole-body PS, PB, and NET did not differ (all, *P* > 0.5) between treatments (Fig. 4a-c).

A treatment-by-condition interaction (*P* = 0.001) was observed for whole-body PS such that postprandial PS was greater than postabsorptive PS for WHEY and EAA + W (*P* = 0.008 and *P* = 0.001, respectively), but not for MEAL (*P* = 0.6). Postprandial PS for EAA + W was 14.3 g/180 min (10.6, 18.4; *P* = 0.001) and 19.7 g/180 min (15.8, 23.6; *P* = 0.001) greater than both WHEY and MEAL respectively, and postprandial PS for WHEY was 5.2 g/180 min (1.3, 9.1); *P* = 0.006) greater than MEAL **(**Fig. 4a). A treatment-by-condition interaction (*P* = 0.011) was observed for whole-body PB such that postprandial PB was lower than postabsorptive PB in all treatments. In the postprandial state, PB was − 7.8 g/180 min (− 12.3, − 3.2); *P* = 0.001, and − 9.1 g/180 min (− 13.7, − 4.5); *P* = 0.001) lower for EAA + W and MEAL, respectively, versus WHEY, but did not differ between EAA + W and MEAL (*P* = 1.0, Fig. 4b). A treatment-by-condition interaction (*P* = 0.001) was observed for NET such that postprandial versus postabsorptive NET was increased in all treatments. Postprandial NET was 22.3 g/180 min (20.2, 24.4; *P* = 0.001) and 18.4 g/180 min (16.3, 20.5; *P* = 0.001) greater in EAA + W than WHEY and MEAL, respectively, and was also 3.9 g/180 min (1.8, 6.0; *P* = 0.001) greater in MEAL versus WHEY (Fig. 4c).

Changes in whole-body PS were 15.8 g/180 min (9.8, 21.9; *P* = 0.001) and 19.4 g/180 min (14.8, 24.0; *P* = 0.001) greater for EAA + W than WHEY and MEAL, respectively, but did not differ between WHEY and MEAL (*P* = 0.09, Fig. 4d). Reductions in whole-body PB were 6.3 g/180 min (− 11.5, − 1.18; *P* = 0.02) greater for EAA + W than WHEY and 7.7 g/180 min (− 11.9, − 3.6; *P* = 0.002) greater for MEAL than WHEY, but did not differ between EAA + W and MEAL (*P* = 0.37, Fig. 4d). As a result, change in NET was 22.1 g/180 min (20.5, 23.8; *P* = 0.001) and 18.0 g/180 min (16.5, 19.5; *P* = 0.001) more positive for EAA + W than WHEY and MEAL, respectively (Fig. 4d**)**. Also, change in NET was 4.2 g/180 min (2.7, 5.6; *P* = 0.001) more positive for MEAL than WHEY (Fig. 4d**)**. Changes in whole-body PS relative to EAA intake were 0.61 g/180 min/g EAA (0.32, 0.90; *P* = 0.001) and 0.77 g/180 min/g EAA (0.48, 1.07); *P* = 0.001) greater for EAA + W than WHEY and MEAL, respectively, but did not differ between WHEY and MEAL (*P* = 0.32, Fig. 4e).

**Fig. 5:**
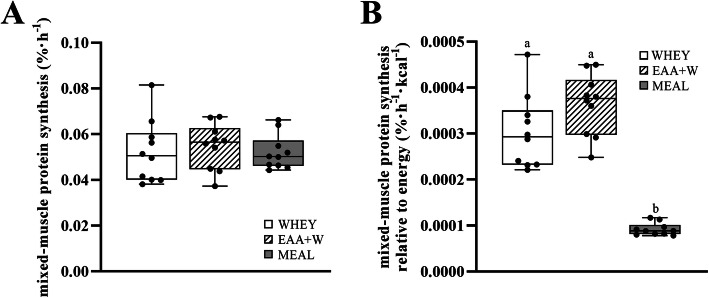
**a** Mean ± SD (*n* = 10). Mixed-muscle protein synthesis responses to whole-body exercise plus post exercise recovery feeding with WHEY, EAA + W, and MEAL intake during energy deficit. No difference between treatments (*P* = 0.68). **b** Mean ± SD (*n* = 10). Relative mixed-muscle protein synthesis responses, expressed relative to study treatment energy, to whole-body exercise plus post exercise recovery feeding with WHEY, EAA + W, and MEAL intake during energy deficit. Different lowercase letters indicate difference between treatment (both, *P* < 0.05)

Postabsorptive phenylalanine hydroxylation did not differ between treatments (all, *P* > 0.5). Postprandial phenylalanine hydroxylation was greater than postabsorptive phenylalanine hydroxylation for EAA + W and WHEY (treatment-by-condition, *P* = 0.001), but not MEAL (*P* = 0.5). Postprandial phenylalanine hydroxylation was 0.07 μmol/kg/min (0.04, 0.09; *P* = 0.001) and 0.09 μmol/kg/min (0.07, 0.11; *P* = 0.001) greater for EAA + W than WHEY and MEAL, respectively, and 0.02 μmol/kg/min (0.00, 0.04; *P* = 0.044) greater for WHEY than MEAL (Fig. 4f**)**. Mixed-MPS did not differ (*P* = 0.68) between WHEY, EAA + W, and MEAL (Fig. 5a). Mixed-MPS relative to the energy content when consuming WHEY and EAA + W did not differ (*P* = 0.063), but were 0.00021%/h (0.00013, 0.00029; *P* = 0.001) and 0.00027%/h (0.00022, 0.00033; *P* = 0.001) greater, respectively, than MEAL (Fig. 5b).

### Amino acid and insulin concentrations

**Fig. 6:**
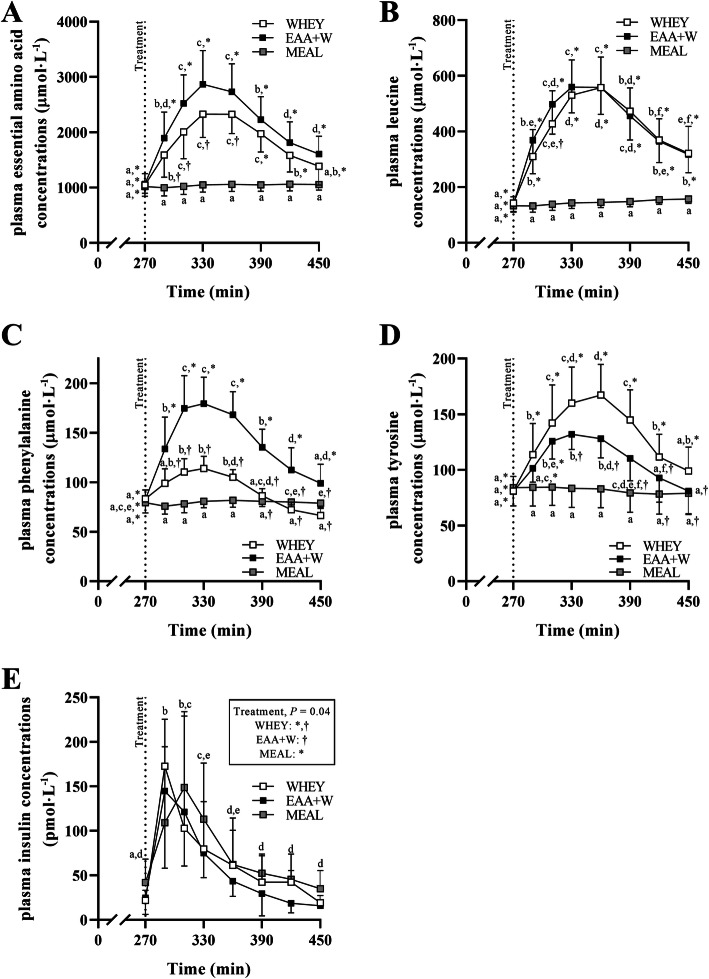
**a** Mean ± SD (*n* = 10). Plasma essential amino acid concentrations after WHEY, EAA + W, and MEAL intake during energy deficit. Different symbols indicate post hoc difference (all, *P* < 0.05) between treatments within a time point. Different lowercase letters indicate post hoc difference between time points within a treatment (all, *P* < 0.05). **b** Plasma leucine concentrations after WHEY, EAA + W, and MEAL intake during energy deficit. Different symbols indicate post hoc difference (all, *P* < 0.02) between treatments within a time point. Different lowercase letters indicate post hoc difference between time points within a treatment (*P* < 0.03). **c** Plasma phenylalanine concentrations after WHEY, EAA + W, and MEAL intake during energy deficit. Different symbols indicate post hoc difference (all, *P* < 0.02) between treatments within a time point. Different lowercase letters indicate post hoc difference between time points within a treatment (*P* < 0.05). **d** Plasma tyrosine concentrations after WHEY, EAA + W, and MEAL intake during energy deficit. Different symbols indicate post hoc difference (all, *P* < 0.04) between treatments within a time point. Different lowercase letters indicate post hoc difference between time points within a treatment (*P* < 0.03). **e** Mean ± SD (*n* = 10). Plasma insulin concentrations after WHEY, EAA + W, and MEAL intake during energy deficit. Different symbols indicate difference between treatment independent of time point (*P* = 0.04). Different lowercase letters indicate difference between time points independent of treatment (*P* < 0.01)

A treatment-by-time interaction (all, *P* = 0.001) was observed for plasma EAA, leucine, phenylalanine, and tyrosine concentrations. EAA, phenylalanine, and tyrosine concentrations increased (all, *P* < 0.05) over time until peaking between 330 and 390 min for EAA + W and WHEY. EAA and phenylalanine peak concentrations were greater for EAA + W, followed by WHEY, and then MEAL (all, *P* < 0.05, Fig. 6a, c). Tyrosine peak concentrations were greater for WHEY followed by EAA + W, and then MEAL (all, *P* = 0.05, Fig. 6d). Leucine concentrations increased (all, *P* < 0.05) over time until peaking between 330 and 390 min similarly for EAA + W and WHEY and were greater for EAA + W and WHEY than MEAL (all, *P* < 0.05, Fig. 6b). Insulin concentrations increased and peaked between 270 and 330 min in all treatments (main effect time, *P* = 0.001, Fig. 6e). There was also a main effect of treatment (*P* = 0.04) such that insulin concentrations for MEAL were greater than EAA + W (*P* = 0.03, Fig. 6e). There were no differences in insulin concentrations between MEAL and WHEY (*P* = 0.26) or EAA + W and WHEY (*P* = 1.0, Fig. 6e). EAA and phenylalanine concentration iAUCs were greater for EAA + W, followed by WHEY, then MEAL (all, *P* < 0.01, Table 6). Tyrosine iAUC was greater for WHEY, followed by EAA + W, then MEAL (all *P* < 0.01, Table 6). Leucine iAUC was similar between EAA + W and WHEY, but both were greater than MEAL (both, *P* < 0.01, Table 6). Insulin concentration iAUC did not differ between treatments (*P* = 0.54, Table 6).

**Table 6:** Plasma amino acid and insulin concentrations presented as incremental area under the curve^1^

	EAA + W	WHEY	MEAL
*Incremental Area Under Curve*			
EAA (μmol/L/180 min)	208,148.1 ± 53,066.6 ^a^	149,433.2 ± 40,539.0 ^b^	4588.6 ± 18,990.5 ^c^
leucine (μmol/L/180 min)	54,054.5 ± 10,469.3 ^a^	50,599.2 ± 11,178.9 ^a^	2179.8 ± 3112.2 ^b^
phenylalanine (μmol/L/180 min)	10,617.8 ± 2737.5 ^a^	1780.7 ± 1292.3 ^b^	77.5 ± 1168.3 ^c^
tyrosine (μmol/L/180 min)	5318.0 ± 1715.4 ^a^	9543.2 ± 3118.5 ^b^	-421.4 ± 817.2 ^c^
insulin (pmol/L/180 min)	7806.8 ± 5614.3	5992.1 ± 4042.2	6179.5 ± 5915.9

^1^Values are means ± SD (*n* = 10). Incremental area under the curve for plasma amino acid and plasma insulin concentrations measured during the infusion studies. Different lowercase letter superscripts indicate difference (all, *P* < 0.01) between treatments for each variable detected using repeated measures ANOVA. EAA, essential amino acid

## Discussion

The purpose of this study was to determine the effects of various EAA/protein delivery formats on post-exercise whole-body protein balance in healthy, young adults after a 5 d, 30% energy deficit. Mixed-MPS responses to the combined effects of exercise and recovery feeding were also determined. The primary finding of this work was that post-exercise NET was greatest after EAA + W ingestion. The superior postprandial NET response following EAA + W was related to a greater increase in peripheral EAA, and a greater increase in PS compared to the other treatments. In addition, there was a greater reduction in PB with EAA + W compared to WHEY. Regardless of differences in NET, mixed-MPS was the same across treatments. However, EAA + W and WHEY had a greater anabolic response when normalizing mixed-MPS to total energy intake, suggesting a greater efficiency of these formats in maintaining MPS. The data suggest that consuming high-quality intact protein enriched with free-form EAA elicits enhanced whole-body protein balance compared to the other iso-nitrogenous formats. Therefore, the combined EAA/protein delivery format may be an effective strategy to offset body protein loss during the catabolic stress of energy deficit.

The marked increase in NET after ingesting EAA + W confirms our hypothesis that changes in NET reflect increases in circulating EAA concentrations. These findings extend our previous study [42] which demonstrated greater NET after ingesting EAA-enriched whey compared to an iso-nitrogenous whey-based recovery product. In the current study, greater NET after ingesting EAA + W was due to a robust increase in PS and concomitant reduction in PB. Based upon previous work [42, 43], it is likely that the resultant NET was largely driven by the circulating EAA profiles. The enhanced peripheral EAA concentrations induced by the free-form and whey-derived components of EAA + W provide the required EAA to initially stimulate PS, as well as the non-EAA for a sustained increase of PS [44, 45]. In addition, a dose-dependent inhibition of PB by EAA has been demonstrated in the splanchnic region, independent of any insulin-specific effects [46]. Lastly, the non-EAA component of the EAA + W likely also provides for a greater use of exogenous EAA for PS, rather than their conversion to non-EAA.

Interestingly, reductions in PB were similar between EAA + W and MEAL. The greater reduction in PB with MEAL was likely due in part to the energy in the MEAL (567 kcals) compared to EAA + W (173 kcals). Given the catabolic stress of energy deficit, the substantial exogenous energy supplied by MEAL may have reduced the requirement for endogenous proteins to provide precursor amino acids used for gluconeogenesis and energy yielding purposes [47]. Regardless, NET was greater for EAA + W due to a greater stimulation of PS. This stimulation supports the effectiveness of this EAA/protein format to enhance whole-body protein balance in the post-exercise recovery period during energy deficit. As mentioned, the current whole-body protein turnover data is limited to the postprandial period and should be interpreted within the context an acute, early postprandial response. However, the plasma EAA concentrations support the whole-body protein turnover data, indicating that peripheral EAA concentrations dictate the whole-body kinetic response. The MEAL is therefore a less efficient and less anabolic EAA/protein format compared to EAA + W. Achieving an iso-EAA comparison between these formats would have required more than twice the amount of food and energy (i.e., 1193 kcal) than the MEAL. However, it is important to note that when whole-body protein turnover is normalized to EAA intake, the change in PS is still greatest following EAA + W.

In addition to measuring whole-body protein turnover, our intent was to determine whether the free-form EAA component, or absence of free-from EAA, within each EAA/protein format would impact mixed-MPS. Despite the enhanced peripheral EAA concentrations with EAA + W, mixed-MPS was equally stimulated between treatments during a combined exercise and recovery period. Lack of an effect in mixed-MPS despite differences in peripheral EAA availability is in agreement with our previous work, showing no difference in mixed-MPS responses to post-exercise ingestion of two doses of free-form EAA during energy deficit [9]. While we cannot report the extent to which mixed-MPS was stimulated in the current study, the lack of differences between treatments is likely due to that fact that during energy deficit rapidly absorbed exogenous amino acids, both EAA and non-EAA, are prioritized centrally (i.e., liver, splanchnic region) to meet whole-body amino acid and energy (i.e., provision of carbon skeletons) requirements. This metabolic prioritization during energy deficit is highlighted by the similar mixed-MPS effect across treatments despite significantly lower aminoacidemia in the MEAL condition, which might be expected to result in lower mixed-MPS under normal physiological conditions (i.e., energy balance). Metabolic prioritization is also reflected in the demonstrated changes in whole-body protein turnover between formats despite no difference in mixed-MPS. Regardless of similar absolute mixed-MPS, an important finding was that EAA + W and WHEY resulted in greater mixed-MPS when normalized to total energy intake. The greater synthetic response achieved by the less energy dense formulations highlights the greater energy efficiency of these EAA/protein formats for supporting MPS compared to MEAL.

Practical dietary strategies for attenuating the deleterious effects of unavoidable energy deficits experienced by military personnel during strenuous training or combat operations on whole-body protein balance and MPS remain limited. In addition to the optimal delivery of EAA/protein required to enhance protein kinetics under physiological stress, nutrition within military operational environments requires that protein formats be easy to carry and convenient to consume [15]. For example, while current combat rations are designed to provide adequate nutrition when consumed in their entirety, inadequate intakes result from the limited time to eat and the limited capacity to carry food [48]. Lightweight, eat-on-the-go products provide a practical opportunity to reduce the mass military personnel must carry on their person compared to current combat ration formats. Our results indicate that a formulation of EAA-enriched, low dose whey represents a logistically feasible and easily consumed EAA/protein format which induces whole-body protein anabolism. The enhanced whole-body anabolic response, more efficient MPS response, and the manageable serving size and preparation of the EAA-enriched whey highlights both physiological and practical benefits for this format as an eat-on-the-go supplemental food. Beyond the military setting, this EAA/protein format would be useful for weight-class athletes seeking to maintain protein anabolism during planned periods of energy deficit.

In conclusion, EAA-enriched whey enhanced NET versus iso-nitrogenous amounts of whey isolate and a mixed-macronutrient meal during a 30% energy deficit. NET was achieved through an increase in PS and an attenuation of PB. In addition, EAA-enriched whey resulted in similar mixed-MPS rates as the other treatments, though MPS was more efficient for the given energy intake since similar rates of MPS were achieved. These findings indicate that protein-containing food formats which have a high EAA content and achieve rapid and sustained peripheral EAA concentrations can enhance whole-body protein status and efficiently support MPS during the catabolic stress of underfeeding.

### Disclaimers

The opinions or assertions contained herein are the private views of the authors and are not to be construed as official or as reflecting the views of the Army or the Department of Defense. Any citations of commercial organizations and trade names in this report do not constitute an official Department of the Army endorsement of approval of the products or services of these organizations.

## Data Availability

The datasets used or analyzed during the present study are available from the corresponding author on reasonable request.

## Abbreviations

ANOVA: Analysis of variance
AUC: Area under the curve
DXA: Dual energy X-ray absorptiometry
EAA: Essential amino acids
EAA + W: Essential amino acid-enriched whey
iAUC: Incremental area under the curve
LC: Load carriage
MEAL: Mixed-macronutrient meal
MPE: Mole percent excess
mixed-MPS: Mixed-muscle protein synthesis
MPS: Muscle protein synthesis
PHE: Phenylalanine
E_p_: Precursor enrichment
RM: Repetition maximum
SSA: Sulfosalicylic acid
TTR: Tracer-to-tracee
TYR: Tyrosine
WHEY: Whey
PS: Whole-body protein synthesis
PB: Whole-body protein breakdown
NET: Whole-body net protein balance
